# HIV/AIDS stigma and utilization of voluntary counselling and testing in Nigeria

**DOI:** 10.1186/1471-2458-13-465

**Published:** 2013-05-13

**Authors:** Clifford Odimegwu, Sunday A Adedini, Dorothy N Ononokpono

**Affiliations:** 1 Program in Demography and Population Studies, Schools of Public Health and Social Sciences, University of the Witwatersrand, Johannesburg, South Africa; 2 Demography and Social Statistics Department, Faculty of Social Sciences, Obafemi Awolowo University, Ile-Ife, Nigeria; 3 Department of Sociology, University of Uyo, Uyo, Nigeria

**Keywords:** HIV/AIDS, Stigma, Nigeria, Cross-sectional, Ethnic, Qualitative, VCT

## Abstract

**Background:**

Despite the recognition of stigma as a hindrance to public health treatment and prevention there are gaps in evidence on the relationship between HIV stigma and VCT services utilization in Nigeria. The purpose of this study was to examine a community’s perceptions, feelings and attitudes towards people living with HIV/AIDS and how this is associated with access to utilization of voluntary counselling and treatment in Nigeria.

**Methods:**

A cross-sectional random study of Nigerians, using a mixed-method approach was carried out in two distinct ethnic areas of the country. Both quantitative and qualitative methods (mixed-methods) were used to collect data in Osun State (Yoruba ethnic group) in the South-West and Imo State (Igbo ethnic group) in the South East. Multivariate logistic regression was the model used to examine the association of interest.

**Results:**

It is shown that Nigerian public attitudes to HIV/AIDS and those infected with the disease are negative. The markers for stigma on the overall stigma index are significant predictors of utilization of voluntary counselling and testing. As the sum of negative feelings increases, there is less likelihood to using voluntary counselling and testing (VCT) and vice versa.

**Conclusions:**

Current national efforts at addressing the AIDS pandemic can only be successful when the issue of AIDS is de-stigmatized and is made a critical part of those efforts. One way to do this is through well-designed messages that should be posted in the media, community halls, health centers and other public places aimed at humanizing the disease and those affected and infected by it.

## Background

While the HIV/AIDS pandemic has been inflicting a devastating impact on various sectors of life, one of the major obstacles to its prevention is social stigma. Stigma is defined as an attribute that is deeply discrediting which links a person to undesirable characteristics, thus reducing that individual’s status in the eyes of society [1-6].

It has been indicated that stigmatized individuals are believed to possess some features that convey a social identity that is devalued in a particular social context. In stigma, one group sees the other as abnormal and should be abhorred. Its components include people distinguishing and labelling human differences; dominant groups linking labeled persons to undesirable characteristics; labeled persons are placed in distinct categories so as to accomplish some degree of separation of ‘us’ from ‘them’, and labeled persons experience status loss and discrimination that leads to unequal outcomes [7]. It manifests in avoidance, social distancing, coercion and non-supportiveness, self-stigma leads to reduced or diminished self-esteem. Stigmatization can lead to prejudicial thoughts, behaviors and actions on the part of individuals and groups [8,9].

The stigma and discrimination associated with HIV/AIDS has been conceptualized at two levels: societal and individual. At the societal or cultural level, it manifest in discriminatory laws, policies, popular discourse and social conditions of people living with HIV/AIDS [10]. At the individual level, it takes the form of behaviours, thoughts and feelings that express the prejudice against persons infected with HIV. The primary targets of HIV/AIDS stigma are individuals with HIV and those perceived to be infected.

From the time that HIV was discovered, it has been accompanied with social response of fear, denial, stigma and discrimination. This gives rise to anxiety and prejudice against the groups most affected. Stigma and discrimination have become the tragic consequences of HIV disease [11] while many referred to AIDS stigma as the third epidemic, noting that the challenge faced by many HIV/AIDS programmes remain the problem of stigma and discrimination [8]. Stigma is not new to public health, nor is it unique to HIV/AIDS. It has been associated with a number of diseases such as leprosy, urinary incontinence, and mental illness [12]. Existing studies and commentaries have noted that stigma and discrimination remain a major fact of life for persons living with HIV/AIDS in sub-Saharan Africa [12,13].

The stigma associated with the HIV/AIDS is not a new phenomenon in public health, as many other diseases have also been stigmatized. This association has often limited treatment and prevention of the particular diseases by the groups so stigmatized [9,10]. In the case of AIDS, the nature and intensity of the stigma is shaped by the social construction of the epidemic as God’s vengeance against sinful practices which varies across cultures and national borders. This variation is affected by local epidemiology of HIV, pre-existing beliefs and values surrounding sexuality, disease, gender and prejudices toward specific out groups like commercial sex workers, lesbians etc. These stigmatized groups are usually considered deviant or shameful and as a result are shunned, avoided, discredited, rejected, restrained and or penalized [14-26]. The consequences of this stigma and discrimination of HIV/AIDS targets have been documented [10].

The importance of studying the role of stigma in research for control of the diseases has been acknowledged by international organizations and conferences. Since it hampers society’s ability to respond effectively to HIV/AIDS, understanding and counteracting AIDS-stigma will remain a critical public health issue in the country. For current HIV/AIDS prevention initiatives in Nigeria to be effective, there is need for research to illuminate the cultural context of AIDS-stigma in Nigeria. This should address how stigma occurs, be resisted and identify ways in which stigma contributes to the spread of the diseases as well as individual responses to it. Understanding this has very important sociological and public health implications. From sociological perspective, it can help us to understand how stigma interacts with other social processes and anticipate its antecedents and consequences. From the public health point of view, it is important to know which social groups are most likely to experience stigma and its adverse consequences so that limited public resources can be used in the most effective way [12,23,25,26].

In Nigeria, a cumulated total of 5.3 million cases had been reported with a national HIV prevalence rate of 5.7% and a high level of regional variation in HIV prevalence [27]. A national programme, HIV/AIDS Emergency Action Plan, involving a cocktail of emergency policies has been put in place. The objective of the plan includes increasing awareness and sensitization of general population and key stakeholders to ensuring that laws and policies encourage the mitigation of HIV/AIDS.

The HIV/AIDS Emergency Action Programme (HEAP) is designed to promote a multisectoral participatory response to HIV/AIDS prevention and impact mitigation. It serves as an expression of govermnent’s interest and commitment to a dynamic and proactive response to the problem of HIV/AIDS. This framework identifies over 200 activities, which Nigerian Government intends to pursue [28].

Further, stigma and discrimination have been reported as factors constituting a serious impediment for VCT uptake and HIV prevention in Nigeria [29] and elsewhere [30]. Stigmatization can go a long way in discouraging people from utilization of VCT. For instance, a study by Okoro et al [31] established that people living with HIV and AIDS (PLWHA) tend to experience self and anticipated stigmas due to their weak appearance caused by HIV-related complication, particularly during life before receiving ART. In a study by Johnson [32], about two-thirds of PLWHA did not make their status known to people outside their family because of fear of stigmatization, and about half of these respondents have suffered stigmatization from their spouse. A study conducted in Abuja, Nigeria by Owolabi and colleagues [33] established blame, insults, social isolation, spread of information, rejection, dismissal from working place, isolation from other patients and denial of access to health care as some of the forms of stigmatization and discrimination being suffered by PLWHA. A systematic review done by Mbonu et al [34] established a range of issues as discrimination in care, distancing and isolation as some of the barriers to VCT uptake in the sub-Saharan Africa. In addition, Olagbuji and colleagues [35] established that fear of stigmatization, break in relationship and spread of confidential information are the most important reasons for non-disclosure of status. Yahaya et al [36] also found that discrimination and stigma as well as poverty and ignorance are some of the reasons for the low utilization of VCT in Kwara State of Nigeria.

Unfortunately, there are gaps in evidence on the extent to which stigma affects VCT uptake in Nigeria, and empirical data on this would be especially useful for developing health policy and for current AIDS programme. Empirical research is also needed to address the effect/impact of stigma on persons at risk for HIV infection. Thus, we need cross-sectional research to document the impact of stigma on the decisions to be tested. This study is part of a two-phased survey to investigate the association of stigma with HIV/AIDS prevention, care and treatment activities in Nigeria. This first phase examined the dimensions of HIV-related stigma in the communities and families, and its potential effect on access to voluntary counseling and testing services.

## Methods

### Study area

This was an exploratory study in two states in the country representing two main ethnic groups^a^ These states were chosen for the sake of convenience. These are Osun State in the western part of Nigeria with an AIDS prevalence rate of 4.7 percent, and Imo State in the eastern part of Nigeria with a prevalence rate of 5.4 percent.

In the two states, two urban centers and two rural communities were selected. The urban cities of Osogbo in the state of Osun; and Orlu in the state of Imo with the adjoining rural communities to each of these cities were selected. Each of the urban centers has all the indices of modernization while the rural areas are relatively homogenous with respect to culture, behaviour and perceptions. In cities, there are a number of organizations active in HV/AIDS prevention activities while in rural communities, local governments are involved in the AIDS campaigns to educate the rural people about the disease and its prevention methods.

#### Recruitment and data collection – qualitative data

At the first level of the study, qualitative data collection was done using the tools of focus group discussions, in-depth interviews and key informants. The participants in the qualitative data collection were community members, community leaders, and leaders of faith-based organizations. In each state, there were eight focus group discussions with young and adult community members (including community health care providers). Eight key informant interviews were also conducted with community leaders, traditional rulers and religious leaders in order to obtain information on the community perceptions and opinions about HIV/AIDS pandemic and attitude towards VCT uptakes in the communities. In addition, eight in-depth interviews were conducted with traditional/religious leaders to collect information on religious and traditional beliefs about HIV/AIDS and death due to AIDS as compared to death due to other causes. The size of the focus groups ranged from 6 to 9 of each category selected. Information on the number of participants in each focus group as well as number of interviews conducted in rural and urban areas is presented in Table 1.

**Table 1:** Characteristics of the FGD and interview participants

**Characteristics**	**Imo State (Igbo)**		**Osun State (Yoruba)**	
	Number of FGD*		**Number of FGD**	
	**Rural**	**Urban**	**Rural**	**Urban**
Young men	1 (7)	1 (6)	1 (9)	1 (7)
(Aged 18-34)				
Older men (35+)	1 (8)	1 (7)	1 (6)	1 (7)
Young women	1 (9)	1 (7)	1 (7)	1 (8)
(Aged 18-34)				
Older women (35+)	1 (6)	1 (8)	1 (6)	1 (6)
**Total number of FGD sessions**	**8**		**8**	
	Number of interviews			
Community leaders	2	2	2	2
Traditional/religious leaders	2	2	2	2
**Total number of interviews**	**8**		**8**	

*Numbers of participants in each group are in parenthesis.

The focus group discussions and in-depth interviews centered on community perceptions of, attitudes to stigma, people living with HIV/AIDS etc. Lastly. questions were asked on their awareness of various current/on-going HIV/AIDS prevention programs in the country and willingness to access any of them, especially voluntary counselling and testing and mother-to-child transmission prevention services.

The size of the focus groups ranged from 6 to 9 participants in each category selected. The group discussions were led by trained male and female research assistants, most of whom were graduate students. They were given one-week training in research methodologies involving focus group facilitation and triangulation^b^ The group discussions were conducted in local language of Yoruba for Osun State, and Igbo for Imo State. Though less often, English was used where groups preferred it. The discussions were audio taped and later transcribed.

#### Recruitment and data collection – quantitative data

A home visitation survey was done for the collection of quantitative data. The sample was drawn from the entire population of the two sites broadly representing different social strata. The social and economic status of the resident population was decided based on the types of residential buildings and such socio-economic factors as education, occupation etc. In the case of the two urban sites, the areas were divided into residential districts. In most Nigerian cities, residential demarcation is used to classify the status of the residents. Broadly, there are three types of residential zones in each of the urban areas of the study. They are traditional residential areas inhabited by poor i*ndigenes* of the area; the migrants’ zone inhabited by medium income earners who are mostly migrants to the town and the government reserved estates inhabited by top government officials and *nouve riche* in the town. The migrants are individuals (mostly *indigenes* of the same ethnic group) who migrated to the urban center for civil service appointments. The two rural areas associated with each of the urban sites are homogenous in terms of culture, although different levels of socioeconomic characteristics exist. There was no need to demarcate the area. Hence, the simple random strategy was used in selecting rural respondents.

After enumerating the households by residential zones in the urban areas, we determined an appropriate sample size for each stratum based on probability in proportion to population size. This method permitted us to select a representative sample of respondents from each of the three strata in equal proportions according to the total population in each stratum. In each household, the number of adults present was counted. Where we had more than two adults aged 18 years and above, a lottery method was used to select one of them for the interview.

The purpose of the study was explained to the adults present and their permission sought to conduct the interview. A sample size of 1,200 respondents was expected to be selected but at the end of field work, 987 respondents who gave their (written) consent to participate in the study were interviewed (490 in Osun and 497 in Imo). Respondents were asked about their feelings towards persons living with HIV/AIDS, support for coercive policies, likelihood of avoiding those infected with HIV and beliefs about how the disease is transmitted. Additional questions were asked on their awareness of various on-going HIV/AIDS prevention activities in the country and willingness to access any of the current HIV/AIDS prevention, care and treatment initiatives especially voluntary counseling and testing, and prevention of mother-to-child transmission services in selected tertiary health care institutions in the country.

This approach was easy to adopt because during the qualitative data collection exercise, we established rapport with community members. Moreover, we recruited residents of the areas as research assistants. This greatly facilitated the data collection process.

To measure the various indices of stigma, the following specific questions were asked. The questions were drawn from existing studies on stigma [10,37,38]. Negative/affective feelings toward people with AIDS were measured by asking respondents to rate the extent to which they felt angry at, afraid of and disgusted by people living with HIV/AIDS (PLWHA). Four response categories were provided, for example, “very angry, somewhat angry, a little angry, and not at all angry”. Support for coercive policies was measured by asking the respondents how much they agreed or disagreed that “people with AIDS should be legally separated from others to protect the public health”, and that “the names of people with HIV/AIDS should be made public so others can avoid them”, and that “pregnant women and everybody should be made to undergo compulsory HIV testing”. Four response alternatives were provided viz, “agree strongly, agree somewhat, disagree somewhat and disagree strongly”.

Attributes of responsibility and blame to infected individuals were measured by asking respondents whether they agreed or disagreed that “people who got AIDS have gotten what they deserve or are responsible for their faith or are sexually loose people”. Response alternatives in this case ranged from “strongly agree to strongly disagree”. Avoidant behaviours were also measured by asking respondents to predict their own behavior in each of four different hypothetical situations involving potential contact with a person living with HIV/AIDS. The situations were having a close friend or relative who developed AIDS; having a child attending a school where another student was known to have AIDS; working in an office where a workmate developed AIDS and finding out that the owner of a small neighborhood grocery store had AIDS. For each situation, respondents were offered a number of varieties of response alternatives that represented an avoidant response (examples, not helping to care for a sick friend, avoiding contact with the workmate) or supportive response (e.g., caring for the friend, helping the workmate or treating him the same as always).

Furthermore, the study assessed participant’s beliefs about HIV transmission via such routes as kissing, hugging, mosquito bites, sharing a drinking glass, using public toilets as an infected person or donating blood. Studies have shown a correlation between these beliefs and stigma conditions [31,33].

#### Analysis of qualitative data

In analyzing the transcripts from the focus group discussions, in-depth and key informant interviews, the thematic analysis technique was used to uncover themes and trends. Comments on each aspect of stigma were compared by place of interview. Excerpts of the transcripts were used to complement the quantitative results where possible. Such excerpts are the views expressed by the majority of the discussants. These were also supported with similar views from the in-depth and key informant interviews.

### Analysis of quantitative data

Data entry was done using the Epi-info version 6. Thorough data cleaning was done after the entry by well-trained research assistants. The cleaned data set was then converted into Stata Version 11 software for the descriptive and multivariate analysis. Simple descriptive analysis involving frequencies and cross-tabulations were done and the results are presented below. The quantitative and qualitative data were analyzed separately. The qualitative data excerpts were used to explain some of the quantitative results, where applicable.

#### Statistical analysis

The scale scores for the stigma measures and stigma index were examined using the Cronbach’s Alpha. The principal component analysis was used to construct a stigma index. This index was computed by counting the number of stigma responses each person gave to the items concerning negative/affective feelings, avoidant behavioural intentions, coercive or punitive policies (i.e. quarantine, public disclosure and labelling), attribution of responsibility and blame. Internal consistency for the items was found to be of high level of reliability (0.75) and hence, acceptable. Further analysis to examine the association of stigma measures with utilization of voluntary counselling and testing services was done using the logistic regression model since?because the dependent variable is whether one uses VCT or not. The Logistic regression models were developed in such a way that the models could predict the likelihood of utilizing voluntary counselling and testing services for HIV status in the study locations, after controlling for measures of stigma and socio-economic characteristics.

### Ethical consideration

Permission for this pilot study was obtained from the Ethical Review Board of the Obafemi Awolowo University, Ile-Ife, Nigeria while one of the authors was a staff member there. Survey and qualitative interviews were done upon obtaining written informed and voluntary consent of the participants after the purpose of the study has been explained to them.

## Results and Discussion

Majority of the respondents were from the urban areas. The Igbo constituted more than half of the respondents (55%). Fifty-six percent were females. There were more respondents with tertiary level of education (46.5%), followed by those with secondary level of education (33%). There were also many catholics and protestants (Table 2).

**Table 2:** Percent distribution of respondents by their characteristics, Nigeria

**Characteristics**	**Percent (N = 987)**
Mean age	31.8 years
Place of Survey	
Urban	60.3
Rural	39.7
Ethnic Group	
Igbo	55.0
Yoruba	45.0
Sex of respondents	
Male	42.5
Female	57.5
Education Level	
None	4.2
Primary	16.5
Secondary	32.9
Tertiary	46.5
Religion	
Protestant Orthodoxy	35.9
Roman Catholic Church	46.0
Islamic Religion	14.9
Traditional Religion	3.4
Married Status	
Married	50.4
Single	45.5
Separated/Divorced/Widowed	4.1
**% ever migrated**	35.2
Access to Media	
% own radio	90.4
% TV	81.7
**Frequency of reading newspapers**	
Very often	22.4
Often	29.5
Rarely	32.1
Never	15.7
Don’t Know	0.4
**Frequency of Listening to Radio**	
Very often	40.1
Often	46.5
Rarely	11.2
Never	0.4
Don’t Know	
**Frequency of TV Watching**	
Very often	37.3
Often	34.9
Rarely	22.8
Never	4.6
Don’t Know	0.4

At the time of the suvey, half of the respondents were in marital union. About one-third of respondents have had experience of migration. There is a widespread ownership of media (radio and television). One-third of respondents rarely read newspapers possibly because of the cost of purchase while radio listening is frequently done, so also watching television. The mean age of the respondents was 32 years with a standard deviation of 12.9.

### Beliefs about HIV/AIDS transmission

There is a high level of awareness of HIV/AIDS (95%) and of methods of prevention of HIV/AIDS, a reflection of success of years of information, education and communication (IEC) activities. The reported primary method of prevention is being faithful to one’s partner (67%). Other methods mentioned include condom use (52%), sexual abstinence (46.2%) and avoiding multi-sexual partners. In terms of non-sexual methods of prevention of HIV infection, two-thirds of the respondents mentioned avoidance of sharing of sharp objects (such as needles) with an individual and avoiding blood transfusion (34%).

There are still misconceptions about HIV routes of transmission. One-fifth of the respondents believed that HIV can be contracted from mosquito bites while 19 percent believed that HIV transmission could be by sharing food with an infected person, an indication that they believe that physical contact with a PLWHA can lead to infection. These misconceptions certainly present challenges to IEC activities. It was well understood by the majority of the respondents that a healthy-looking person can be HIV-positive. There is a high awareness of mother-to-child transmission of HIV (85%) and this could be contacted during pregnancy (86%), breast-feeding (66%) and during delivery (56%). About two-thirds have discussed HIV prevention with their partners in the preceding 12 months to the survey (Table 3).

**Table 3:** Percent distribution of respondents’ beliefs about knowledge on HIV/AIDS transmission in Nigeria

**Responses**	**Percent, (n = 987)**
% Aware of HIV/AIDS^1^	94.9 (937)
% Aware of HIV prevention^2^	84.2 (832)
**Mentioned Prevention Methods**	
Fidelity	66.9 (661)
Condom use	52.2 (516)
Sexual abstinence	46.2 (456)
Avoid sex with prostitutes	36.4 (359)
Avoid sex with one with many partners	20.7 (204)
Avoid sex with multi-partners	18.6 (184)
Avoid sex with homosexuals	18.6 (184)
Avoid sex with drug users	8.2 (81)
**Non-sexual methods of prevention**	
Avoid sharing sharp instruments	60.6 (599)
Avoid blood transfusion	34.0 (336)
Avoid kissing	14.2 (140)
Avoid injection	9.1 (90)
Avoid mosquito bites	9.6 (95)
Seek protection from traditional healers	2.7 (27)
**Other HIV-related Beliefs**	
% Chances of reducing HIV infection by sticking to one partner	59.9 (592)
% HIV can be gotten from mosquito bites	23.1 (228)
% One can get HIV by sharing food with a person infected with AIDS	18.5 (183)
% Reduce chances of infection by using condoms every time sex is had	59.9 (592)
% Healthy-looking person can be HIV positive	78.7 (778)
% Aware of persons living with HIV/AIDS	45.5 (449)
% Aware of mother-to-child transmission of HIV	84.5 (835)
% Mother-to-child transmission during pregnancy	86.0 (850)
% Mother-to-child transmission during delivery	56.2 (555)
% Mother-to-child transmission during breastfeeding	65.7 (649)
% Ever discussed HIV prevention with partner	61.3 (606).

^1^Percentage of respondents who reported they were aware of HIV/AIDS.

^2^Percentage of respondents who reported awareness of mode of HIV transmission and prevention.

### Perceptions of and attitudes to HIV-related Stigma

The survey instrument assessed the multiple facets of AIDS stigma. A significant majority of respondents manifested various facets of AIDS stigma.

#### HIV-related stigma

The survey tried to assess multiple facets of HIV/AIDS stigma in Nigeria. Many of the participants said they would be disgusted toward persons living with HIV/AIDS. Others said they would be afraid if they see anybody who is sero-positive. The Igbo respondents expressed more of the feeling of anger and disgust than the Yoruba respondents. Participants from both ethnic groups were afraid of HIV-positive individuals because.

“*The disease disfigures the person. The person will eventually die… In fact you are actually seeing a living ghost. What scares us most is the fatal nature of the diseases. So when we hear that somebody is infected or we see one, we are afraid because the person will die sooner or later*” (FGD, Younger Females).

In terms of support for coercive policies of legal isolation, mandatory testing and public labelling of infected individuals, FGD and in-depth interview participants supported these. They reasoned that taking this hardline approach is to make sure that “*They will not go about infecting people*”. Some of the participants narrated a story of individuals who have been diagnosed positive and went about infecting others because they believed that:

“*Since someone infected them, they too have to infect others, and not die alone”.*

However, respondents of Igbo ethnic origin said they are opposed to it , because it would heighten isolation and increase depression for the infected and will bring shame and ridicule to his family, community and friends. There is recognition among the participants that most families may have infected individuals but would not like the public to know in order to protect the family name.

Most of the participants also expressed the view that sero-positive individuals are responsible for their infection. This view does not differ by ethnicity and sex of the respondents. There is a common view that infected individuals are sexually promiscuous. The common view expressed by discussants was that:

“Sex is the sweet thing that is bitter. It is the infected person’s irresponsible and promiscuous behaviour. If s/he is not loose, how did s/he come about the infection..?”

### Consequences of stigmatization

The participants also indicated the issue of name-calling, social distancing, isolation and depression as markers of HIV/AIDS stigma.

“*Disclosure of HIV status can lead to public labeling. People, friends and relations will abandon you. If you were popular, your best friends will dissociate from you once you are diagnosed to be positive. A baby was recently diagnosed to be positive in the hospital, the father became depressed and vowed never to touch the child*” (Educated Female, age 30-35; IDI).

“*The stigmatized person will feel lonely and loneliness can cause mental problem – depression and suicidal attempts. You will just discover nobody wants to talk to him or her. The problem of isolation and abandonment worsen the situation of being positive*” (Educated Male, age 40, IDI).

A group of participants agreed that

*“Stigmatization can lead to concealment of status so that people will not know. This will deny early medical attentions. Concealment of status is one of the reasons why the infection cannot be controlled. A lot of people have the disease but do not want to declare publicly their status for these fears”.* (FGD, Older Women)

There is awareness of the impact of stigma on economic progress. Participants noted that there will be less patronage of a trader or shop owner infected. Income will be spent on treatment and consultation. This diminishes income and affects the ability of breadwinners to meet other needs of the family or of the individual. If a married individuals, other family responsibilities, such as their children’s education will suffer.

### Negative/affective feelings towards PLWHAs

It is shown in Table 4 that more than two-fifth of the respondents felt ‘very’ or ‘somewhat angry’ or ‘disgusted’ toward persons living with HIV/AIDS, and more than half were afraid of them. Among the Igbo, more than half would feel ‘angry’ and ‘disgusted’ toward people living with HIV/AIDS while equal percentage of respondents from both ethnic groups were afraid of them because:

*The disease disfigures the person. And the person will eventually die… In fact you are actually seeing a living ghost. What scares us most is the fatal nature of the diseases. So when we hear that somebody is infected, we are afraid because the person will die sooner or later” .*(FGD, Younger Females)*.*

**Table 4:** Ethnic differentials in levels of stigma measures among the respondents, Nigeria

	**Ethnic Group**		
**Stigma Measures**	**Igbo (East)**	**Yoruba (West)**	**Total**
**Negative Feelings**			
% Very angry	56.4	32.0*	45.3
% Very Disgusted	53.5	43.0*	49.1
% Very afraid	56.3	56.3	56.3
**Coercive Attitudes**			
% Quarantine PLWHAs	67.0	76.9*	71.8
% Mandatory HIV testing	78.3	93.9*	85.3
% Public Labeling	50.8	39.2	47.1
**Attribution of Blame**			
% Gotten what they deserve	51.8	63.2	57.3
% Sexually loose people	35.6	41.4*	38.3
% Responsible for their illness	55.7	55.0	55.4
**Avoidant Behaviours**			
% Avoid caring for an infected relation	53.9	33.9*	55.9
% Avoid infected shopkeeper	83.0	86.5	84.8
% Avoid infected workmate	72.7	61.6*	67.4
% Avoid child in school of an infected pupil	43.0	55.9	53.4
**Symbolic Contact and Interaction: Comfortable with PLWHA in**			
Shaking hands	43.5	58.5	50.8
Using same plate	15.8	41.3	28.1
Working in same place	41.9	55.6*	48.6
Hugging	16.1	36.3*	25.9
Kissing	5.5	10.2*	7.8
Sharing toilet	14.8	27.1*	20.8
**Attitude to an infected partner**			
Caring for him/her	67.7	58.3*	63.1
Eat with him/her	23.4	43.4	33.1
Sleep together	17.8	41.4*	29.3
Sit and chat together	61.5	59.5	60.5
Walk and move together	55.8	54.5	55.2

* Significant at p <0.05.

Some of the respondents agreed that the society expresses anger to infected individuals because they are seen as being responsible for the infection due to their:

‘*loose life and hence bring shame and ridicule to their families”.*

### Support for coercive policies

Table 4 shows that seven out of every ten of the surveyed respondents supported/held the view that people living with HIV/AIDS should be quarantined; 85.3 percent supported mandatory HIV testing irrespective of the fundamental human rights of individuals and 47 percent supported public labelling/disclosure of infected individuals. There is variation across ethnic divide. Most of the Yoruba respondents also supported quarantining infected individuals. According to these participants, the reason for this support is to make sure that:

“They will not go about infecting other people. This is because an infected person may decide to infect others so that he or she will not die alone.”

In contrast, the Igbo respondents were opposed to quarantine infected individuals because*:*

“it would heighten isolation and depression for the infected and will bring shame and ridicule to his family, community and friends, thereby entrenching stigmatization”.

However, a religious leader noted in the interview that in relation to coercive policies of quarantine or public labeling that:

“*They should not be isolated or quarantined. They should be encouraged because those infected need support from their family, friends, and even the church. They should be educated on what to do or not to do now they are infected. We will be in a position to know the level we can be of assistance to them. No, they should not be quarantined or publicly labeled.”* (Religious leader, IDI)

The majority of participants agreed that blame should not be attributed to persons living with HIV/AIDS because sexual intercourse is not the only means of infection. They noted that the virus can be contacted through other sources.

More than two-fifths of the respondents endorsed public labelling of infected individuals so that:

*“other people should be aware of them and avoid any contact that should expose them to risk of infection”*.

This differs by ethnicity: Half of the Igbo respondents supported this view, compared to 39 percent of the Yoruba respondents. There is noticeable pattern of inconsistency as some of the respondents would not want an infected relation’s name to be made public so that the family name will not be *“*tarnished”. There is recognition here that most families may have infected individuals but would not like the public to know in order to protect the family name. This inconsistency may be an effect of sampling variability or possibly lack of understanding of the question.

### Attributing responsibility and blame

Also over half of the respondents agreed that PLWHAs deserve their illness because they are personally responsible for the sickness (55%). There is not much difference between the two ethnic groups (Table 4). Also, over one-third of the respondents agreed that infected individuals are “sexually loose” people. More Yoruba respondents agreed to this view than the Igbo respondents (41.4% vs 36%). However, during the in-depth interviews and focus group discussions, more of the Yoruba participants spoke against this view arguing that:

*“It is not only through sexual intercourse that one can get the disease”*. But among the Igbo *“it is the sweet thing that is bitter. It is his or her irresponsible behavior. If s/he is not loose, how did s/he come about the infection?”.*

As can be seen here, in both ethnic groups, these two views are common. What is clear in the qualitative data is the fact that in Nigeria, significant number of people still sees HIV infection as an artifact of sexual promiscuity and hence infected individuals should be held responsible and be blamed.

### Avoidant behavioral intentions

While some respondents agreed that they should care for the infected and affected individuals, the majority said they would also avoid a shopkeeper who is infected (84.8%). More than two-third said they would avoid a workmate who is HIV-positive and 53% of the surveyed respondents would withdraw their children from a school where a mate or teacher is infected (Table 4). This pattern of negative responses is borne from the persistent belief that one can contact the disease if there is interaction with an infected person. Many participants in the qualitative interviews felt that it might be a result of fear:

“*You see, an infected person is as good as dead. So, interacting with him will be as if you are playing with a ghost. There is the fear in you that this person will soon die and this fear translates into some of your behaviours towards that person*”.

### Symbolic interactions

In terms of symbolic interactions with persons living with HIV/AIDS, Table 4 also shows that 51 percent claimed they would be comfortable shaking hands with an infected person (59% in Yoruba and 44% in Igbo). Close to one-third would be comfortable using the same plate to eat, hugging an HIV-positive person, and one-fifth would share a toilet with a PLWHA. There is a significant ethnic variation in the pattern of responses with more Yoruba in favour of doing these things than the Igbo. The Yoruba show a more positive attitude to interaction with HIV-positive individuals.

Some respondents agreed that they should care for the infected and affected individuals, 56 percent would avoid caring for an infected close relation (54% among the Igbo and 34% of the Yoruba respondents). The majority said they would also avoid a shopkeeper who is infected with HIV. Other avoidance behaviours are also expressed such as if a workmate is positive and also their child will be withdrawn from a school where a classmate is infected.

For interactions with an infected partner, about one-third said they would be comfortable caring for an infected partner, sitting and chatting together with him or her. More of the Igbo respondents would care for their partners than the Yoruba but more of the respondents would eat with an infected partner (43%), sleep together (41%). The concept of ‘sleeping together’ was confusing to the respondents. Most of the respondents that answered in the affirmative thought it meant sleeping on the same bed. When it was clarified that it meant having sex, almost all rejected that, insisting that they would stop once their partner is diagnosed positive. Most female respondents reported that they would not have sex even if the HIV-positive partners use condoms. On the other hand, men reported that they also would not have sex with their infected partners. While female respondents reported they would have sympathy for their infected partners, their male counterparts said they would terminate the relationship or if not positive, marry another woman/partner.

The female participants in the focus group discussions commented thus:

*“If my partner is infected, I will test to see if I am too. I will not abandon him if we are properly married. I will care for him but we shall not have sex again or bear children because there is no need for bring a child that will lose both parents soon*”.

A male participants noted:

*“Ah, my partner to be infected? I will check myself too. If I am not, then I will deal with her; terminate the relationship and throw her out of my house. It shows she has all along being cheating me. She will be made to pay for her sins*”. (Older Men in an FGD)

Married respondents said that they would take steps to protect their children if they (respondents) realize that they are positive and argued that:

*“If we are positive and knowing that it can be transferred from mother to child, we will stop sexual activity and reproduction. If we have had babies, fine, we will screen them and if any of them is positive, we will as much endeavour to access treatment and care*”.(excerpts from male and female FGDs)

The female participants in both the FGDs and IDIs expressed a higher degree of empathy for a positive spouse, than male participants:

*“I will take care of him and take him to the hospital …. We should not run away from AIDS victims because they are still human beings. We will interact with the person, providing all the necessary emotional and physical support to make him happy…. If we abandon an AIDS patient, he can die of depression”.* (Female aged 35-40, IDIs)

While some men expressed similar empathy, majority in both FGDs and in-depth interviews disagreed. For some of the men, they said:while others responded thus

“We will continue to watch her till she dies….; The person has become a masquerade that everybody will be running away from. One cannot move nearer to her”.

*“If my wife gets the disease, she will see ‘pepper’. I will disown and disgrace her. I will use all means to discipline her. She is a disgrace. She must explain how she got the infection*”. (Male aged 40-45, IDI)

The gender differences are obvious here. Female respondents showed more positive feelings to infected individuals than their male counterparts.

However, in terms of sexual intercourse with an infected partner, all the respondents irrespective of the ethnic group or sex, rejected that insisting that once their partner is found positive:and for the females:

“*that is the end of the relationship. I will not have sex with him*”. (Male aged 35-40,IDI),

“*I will leave him and look for another person to marry” “If we have not had sexual contact, I will call it quits*”. (Female aged 35-40, IDIs)

Another female respondent said:

*“I will leave him if confirmed once we are not married but if we are married I will restrict him from having sexual intercourse with me. I will not allow him to have sex with me again…”.* (Female aged 35-40, IDIs*)*

### Stigma and utilization of voluntary counseling and testing services

The underlying assumption of this study is that stigmatization of HIV/AIDS and people living with HIV/AIDS will hinder access and use of voluntary counselling and testing for HIV status and or any other AIDS prevention, care and treatment programs. In other words, individuals will not respond positively to current multi-sectoral approach to HIV/AIDS prevention initiatives in Nigeria because of the fear and stigma associated with HIV. Hence, in this study we sought to examine the association of stigma with utilization of HIV voluntary counselling and testing (Table 5).

**Table 5:** Logistic regression showing the likelihood of utilizing voluntary counselling and testing services for HIV status, controlling for socio-economic characteristics and measures of stigma, Nigeria

**Control variables**	**Intention to use VCT services**	**95% Confidence interval**	
**Place of residence**		**Lower level**	**Upper level**
Rural	RC		
Urban	1.81	0.489	1.350
**Ethnic Group**			
Igbo	RC		
Yoruba	1.04	0.597	1.813
**Sex of Respondents**			
Male	RC		
Female	0.60*	0.391	0.9348
**Education Level**			
None	RC		
Primary	2.45*	1.3061	4.6023
Secondary	1.86*	1.0592	3.2717
Tertiary	1.49	0.8627	2.5989
**Marital Status**			
Currently Married	RC		
Single	2.30*	1.289	4.117
**No media access**	0.53*	0.300	0.927
**Beliefs about HIV routes**			
Misconception	0.475*	0.2744	0.8224
**Stigma Measures**			
Negative feelings	0.608*	0.4779	0 .7739
Coercive policies	0.825*	0.7300	0 .9323
Attribution of blame			
No	1.144*	1.029	1.272
Avoidant Behaviour**			
No	1.230*	1.277	1.340
Overall Stigma Index	0.924*	0.8759	0.9744
LR Chi2 (21)	74.42; Prob > chi2 = 0.000		

** Reference categories are in the reverse, *p < 0.05.

There were nine variables found to be significant in this model. Urban respondents were more likely to express an intention to utilize VCT services than the rural respondents. The Yoruba respondents also showed more likelihood to use VCT than their Igbo counterparts. The educated respondents of different categories expressed more likelihood to use VCT services than the reference group. Conversely, female respondents were less likely to use HIV/AIDS prevention services than their male counterparts, and other religious groups were less likely to intend to use VCT services than the reference group of protestants. This seems surprising because in Nigeria, the Catholic Church is more deeply involved in care and support programs for HIV patients than other religious groups. In addition, the odds of expressing intention to use VCT services among the unmarried was 2.3 compared to the married respondents.

The major concern of most of the married participants/respondents is how their partner would react if they are tested and if found positive. Female respondents were especially afraid of the consequences if found to be HIV-positive. There were expressed fears of abandonment and of being ostracised by the family and community. Their male partners would deny being responsible for passing on the infection. In most cases, the woman is abandoned and even punished by the community. Respondents that had no access to the media are less likely to use VCT, possibly because they have not heard of the existence of such health services.

In terms of impact of the HIV stigma on the likelihood of women accessing the use of voluntary counselling and testing services, it is found that as the score for negative feelings increases, there is less of a likelihood that a person will use the VCT. The same response pattern is observed in the case of coercive policies. It is also revealed that those who would not attribute blame and responsibilities reported more likelihood of using voluntary counseling and testing services than otherwise. Respondents in this survey who would not adopt avoidant behaviours are more likely to utilize VCT than those who report avoidant behaviours. The combined influence of stigma measures is more likely to affect voluntary counselling and testing utilization.

Further, this study provides useful and challenging important insights into the extent and role of HIV/AIDS stigma on access and utilization of HIV/AIDS prevention services in the country. There are overt and covert expressions of HIV stigma. The punitive aspects of HIV/AIDS stigma were reported to include isolation of infected individuals, mandatory testing and public labeling/disclosure. It is disturbing that seventeen years after the first HIV case was identified in Nigeria, two-thirds of those surveyed have negative feelings towards persons living with HIV/AIDS [PLWHA], and more than two-thirds support coercive/punitive policies against the PLWHA.

Moreover, there were reported covert forms of stigma as one-quarter of the surveyed respondent felt comfortable having any direct interactions or symbolic contact with PLWHAs. These feelings could become manifest into avoidance behaviours. As this study shows, eight out of ten persons would avoid infected shopkeeper, more than two-third would avoid infected workmate and 56 percent would withdraw their children from a school being attended by an HIV positive child or teacher.

The study also shows that misconceptions about HIV/AIDS can increase the AIDS related stigma. Misconceptions arose from the fact that people see heterosexuality as the only route of infection and that it can be contacted via kissing, hugging an infected persons and by mosquito bites. More than half of the surveyed respondents reported that PLWHA deserve what they got in being infected while slightly more than one-third see them as “loose people” (people who are sexually promiscuous). This means that PLWHAs will be subjected to greater stigma when they are perceived to be personally responsible for their situation. This is a big challenge to current efforts at addressing HIV/AIDS pandemic in Nigeria and public health education campaigns. The implication of these findings is that while public health campaigns have helped to create knowledge of HIV/AIDS, it has not been successful in convincing the public that HIV can be contracted via other routes. Thus, AIDS education in Nigeria should involve giving clear information about how HIV is and is not transmitted so that the prevailing misconceptions can be overcome. The need to educate rural population on various routes of HIV transmission should be intensified. Some church leaders expressed readiness to be trained as peer educators to reach out to their congregations/religious adherents. The faith-based organizations are unexplored and under-used resource for HIV/AIDS prevention, care and treatment in Nigeria.

Gender differences were obvious in this analysis. Female gender showed more positive feelings to infected individuals than the male respondents. Also more of the women support coercive policies than men. More women attribute blame to the infected person than do men. In symbolic interactions, the male respondents show more willingness to interact with HIV-positive individuals than women. These differences are not significant but they contradict earlier studies, which tend to infer that men show more negative reactions to PLWHAs than women. However, in the qualitative data, the women expressed more willingness to interact with an infected partner than an infected workmate, and to show more sympathy than men. Generally, most respondents would interact with their HIV-positive partners than with an infected workmate.

Another significant finding is the manifestation of ethnic differential in AIDS stigma between the Yoruba in the Western part of Nigeria and the Igbo in the East. The different levels of socio-economic development could account for these ethnic differentials. The Western region of Nigeria is the most advanced socio-economic hub of Nigeria in terms of infrastructure, education and NGO activities. Since the discovery of the pandemic in Nigeria in 1986, NGO activities (both national and international) have been concentrated in the western part of the country, especially Lagos and Ibadan. The wide gap that this study found in the level of perception and attitude by the Yoruba and Igbo could also be attributed to this disparity. However, their support for quarantine and mandatory testing for HIV may be explained by the fact that a broad spectrum of the bloc was not interviewed. However, this may be a result of sampling variability because the two areas selected do not constitute the representative areas of Yoruba and Igbo ethnic groups. The sample could have been biased as a result.

## Conclusions

There are recommendations implicit in this study:

First recommendation is that there should be a deliberate re-orientation of media practitioners who in most cases contributed to the stigmatization of the disease. An opinion leader in one of the in-depth interviews observed thus:

*“They [journalists] contributed to this problem. The way they narrated the story of HIV when it started and the picture they showed us still scare us and these have led to the stigma. Before we can accept persons living with HIV/AIDS fully, the media should un-do the harm they have created….”* (A traditional ruler in an interview)

Also, home videos, radio jingles, news media etc should be mobilized to embark on programs to de-stigmatize HIV/AIDS. AIDS education should be integrated into the curriculum of teaching in the country from the primary to tertiary levels.

The second recommendation is that the current policy of some religious groups to demand pre-marital voluntary counseling and testing for HIV should be discouraged because it infringes on the rights of the individuals.

Third, the elites should also be addressing their colleagues and associates. Interacting with infected individuals does not transmit the disease unless an:

*“individual goes out of decency having promiscuous sex all around or getting blood transfusion from mushroom clinics”.* (FGD participants)

Fourth, efforts should also be made to de-urbanize current prevention activities in Nigeria. Locating VCTs in the metropolitan Teaching Hospitals in a situation where the majority lacks resources to utilize health services at the teaching hospitals is counterproductive. Many health units are located in the rural communities. Most of the infected individuals in the urban cities travel back to their home rural areas to ‘die there’ or to consult the local traditional healers or utilize the local health center where fee is low. Funders should make it a definite policy for grant- holders to locate projects in the rural areas. About 65 percent of Nigerians live in the rural areas. In the urban centers, it is easy for infected individuals to access VCT sites but it is difficult for an HIV-positive person to approach VCT sites in rural communities where relationship is primary and individuals are well known. Also, village-level interventions spearheaded by local community associations, religious bodies should be encouraged.

Fifth recommendation is that campaigns against stigma and discrimination should be upfront and clear. This should require the support of the high profile people in the society. The effect of this will be increasing openness about AIDS and limiting the negative effect of stigma on HIV prevention activities. Education campaigns can help people to identify the inaccurate stereotypes about AIDS and replace these with factual information. Contact with PLWHAs can yield significant improvements in attitudes about AIDS illness, as studies have shown that members of the public who are more familiar with an illness are less likely to endorse prejudicial attitudes [39,40]. While external stigmatization is being confronted, internalized stigma should also be addressed. Harmful/Hurtful attitudes about self should be replaced with beliefs that do not undermine the person’s self-esteem.

Sixth recommendation is that, the issue of gender dimensions of stigma should be addressed by directing IEC programs particularly at male individuals who tend to show more negative feelings towards PLWHA than females.

In addition, the findings of this study suggest that many people still associate HIV/AIDS with sexual promiscuity. This belief has continued to cause stigmatic and discriminatory behaviours towards people living with HIV/AIDS. HIV preventive programs that will achieve substantial success must include strategies that educate people that there are various modes of HIV transmission other than sexual intercourse alone. Also, based on the findings that limited media access is associated with less access and usage of VCT services, the role of the media as a means of educating the public and creating awareness about VCT services is vital and should be explored further. The present study established that religious leaders have expressed their willingness to become more involved as peer educators. This finding suggests that religious leaders must be used as a resource for HIV prevention programs in communities.

While much detailed research is needed to unravel the dynamics of stigma, studies that focus on action research are needed to evaluate carefully designed intervention programs to reduce the impact of HIV-stigma and discrimination. Interventions based on current knowledge should be designed and rigorous evaluations of impact done to determine their effectiveness. The results of such studies will increase our knowledge and cognizance of HIV risk factors. The results also should lead to an increase in both the global and national potentials to strengthen HIV/AIDS intervention programmes in Nigeria and globally [9].

### Limitations

The following limitation must be borne in mind while interpreting the findings of this study. The two selected areas – Imo and Osun states may not constitute a representative sample of the areas of Igbo and Yoruba ethnic groups. Nonetheless, the people of Imo state can be regarded as a good representation of Igbo ethnic group because of the similar culture, beliefs and antecedents that people of Imo state share with the rest of the Igbo tribe. The same thing can also be said of the people of Osun state as a good representation of the Yoruba ethnic group.

## Endnotes

^a^The two ethnic groups included in this study – Igbo and Yoruba are the two major ethnic groups in the Southern part of Nigeria and two of the three major ethnic groups in the country. The two groups have different socio-cultural values. In terms of development indicators, the two ethnic groups are the most educated and the most urbanized in the country, with South-west, where Yorubas are concentrated being the most advanced socioeconomic hub of Nigeria in terms of infrastructures, education and non-governmental organizational activities.

^b^Triangulation was used in this study to obtain information on HIV/AIDS disease, attitude to people living with HIV/AIDS (PLWHA) and attitude towards VCT utilization from different standpoints, using qualitative and quantitative approaches.

## Competing interest

The authors declare that they have no competing interest.

## Authors’ contributions

CO was the principal investigator, and writer. SAA participated in the drafting of the manuscript. DNO provided word-processing oversight. All the authors read and approved the final manuscript.

## Pre-publication history

The pre-publication history for this paper can be accessed here:

http://www.biomedcentral.com/1471-2458/13/465/prepub
